# Effect of HMGCR genetic variation on neuroimaging biomarkers in healthy, mild cognitive impairment and Alzheimer's disease cohorts

**DOI:** 10.18632/oncotarget.7797

**Published:** 2016-02-29

**Authors:** Lei Cao, Hui-Fu Wang, Lin Tan, Fu-Rong Sun, Meng-Shan Tan, Chen-Chen Tan, Teng Jiang, Jin-Tai Yu, Lan Tan, Alzheimer's Disease Neuroimaging Initiative

**Affiliations:** ^1^ Department of Neurology, Qingdao Municipal Hospital, Nanjing Medical University, Qingdao, China; ^2^ College of Medicine and Pharmaceutics, Ocean University of China, Qingdao, China; ^3^ Department of Neurology, Qingdao Municipal Hospital, School of Medicine, Qingdao University, Qingdao, China; ^4^ Department of Neurology, Nanjing First Hospital, Nanjing Medical University, Nanjing, China

**Keywords:** Alzheimer's disease, brain structure, HMGCR, neuroimaging, glucose metabolism, Gerotarget

## Abstract

Alzheimer's disease (AD) has become a considerable public health issue. The mechanisms underlying AD onset and progression remain largely unclear. 3-Hydroxy-3-methylglutaryl coenzyme A reductase (*HMGCR*) is a strong functional AD candidate gene because it encodes part of the statin-binding domain of the enzyme, which serves as the rate-limiting step in cholesterol synthesis in all mammalian cells. Here, we evaluated the potential role of *HMGCR* (rs3846662) in AD-related pathology by assessing neuroimaging biomarkers. We enrolled in 812 subjects from the Alzheimer's disease Neuroimaging Initiative dataset. In general, it is possible that *HMGCR* (rs3846662) could be involved in preventing the atrophy of right entorhinal (P=0.03385) and left hippocampus (P=0.01839) in the follow-up research of two years. What's more, it lowered the drop rate of glucose metabolism in right temporal. We then further validated them in the AD, mild cognitive impairment (MCI), normal control (NC) sub-groups. All the results in the MCI groups confirmed the association. The results of our study indicated that *HMGCR* (rs3846662) plays a vital role in AD pathology mainly by influencing brain structure and glucose metabolism during AD progression.

## INTRODUCTION

Alzheimer's disease (AD), characterized by formation of amyloid plaque, neurofibrillary tangles and loss of neurons, is the most prevalent forms of dementia affecting the aging population, and it has become a considerable public health issue [1]. The mechanisms underlying AD onset and progression remain largely unclear. Up to now, the only genetic variant consistently shown to impact AD risk and age at onset was *APOE* [2]. It has been reported that variations in genes associated with cholesterol homeostasis modify the risk of AD progression [2]. 3-Hydroxy-3-methylglutaryl coenzyme A reductase (*HMGCR*) is a strong functional AD candidate gene because it encodes part of the statin-binding domain of the enzyme, which serves as the rate-limiting step in cholesterol synthesis in all mammalian cells [3, 4]. The association of *HMGCR* (rs3846662) with LDL-C levels has been replicated in Japanese Populations [5]. A study identified rs3846662 as a functional SNP in intron13 [6]. Besides, it was in linkage disequilibrium with the SNPs of genome-wide significance and influenced alternative splicing of *HMGCR* mRNA [7]. Compounds having inhibitory effects on the enzyme *HMGCR*, such as statins, are thought to possess anti-inflammatory effects and may have some clinical effects in AD patient [8]. A study has clearly identified *HMGCR* rs3846662 as a potent genetic modifier for AD risk, age of onset and conversion in three cohorts study [4].

Neuroimaging methods increasingly are used as additional outcome measures to explore the link between *HMGCR* rs3846662 and AD. Therefore, this study aims to investigate the involvement of *HMGCR* in the development and progression of AD by studying the influence of *HMGCR* on neuroimaging biomarkers in the three different clinical stages (normal control (NC), mild cognitive impairment (MCI), AD) from the Alzheimer's Disease Neuroimaging Initiative (ADNI) dataset.

## RESULTS

### Demographic and clinical characteristics

The dataset comprised of 48 AD patients (18 women, 75.51±9.23 years), 483 MCI samples (201 women, 72.28±7.45 years) and 281 NC (145 women, 74.51±5.56 years) at baseline in our study (Table 1). In consistent with previous studies, AD patients had the highest frequency of the ε4 allele within *APOE* gene and CN group had the lowest frequency. In addition, the AD patients have worst cognitive function according to the scores of the five neuropsychological scales (Clinical Dementia Rating scale sum of boxes (CDRSB), the Alzheimer's Disease Assessment Scale 11(ADAS11), the Mini-Mental State Examination (MMSE), the Alzheimer's Disease Assessment Scale 13 (ADAS13), the Rey' Auditory Verbal Learning Test (RAVLT) and Functional Activities Questionnaire (FAQ)). Furthermore, no statistical differences were observed among NC, MCI and AD patients when comparing the distribution of the tested SNP allele frequencies in our study.

**Table 1:** Characteristics of eligible studies for meta-analysis

Characteristics	CN		MCI		AD		P*
Age (years)	281	74.51±5.56	483	72.28±7.45	48	75.51±9.23	-
Gender (male/female)	281	136/145	483	282/201	48	30/18	-
Education (years)	281	16.41±2.66	483	15.98±2.82	48	15.73±2.62	0.08
APOE ε4 (0/1/2)	281	204/70/7	483	262/180/41	48	14/25/9	<0.01
CDR-SB	207	0.03±0.13	406	1.44±0.87	47	4.44±1.69	<0.01
MMSE	281	29.07±1.15	483	27.89±1.69	48	22.96±2.03	<0.01
ADAS-cog	281	9.06±4.23	480	15.30±6.65	48	29.80±8.44	<0.01
RAVLT	280	44.83±9.60	483	36.16±10.86	47	22.32±7.84	<0.01
FAQ	281	0.17±0.66	481	2.85±3.99	48	12.6±7.14	<0.01
Hippocampus (mm^3^)	257	7344±895	422	6996±1126	39	5757±948	<0.01
Middle Temporal (mm^3^)	257	20298±2600	422	20186±2735	39	17776±3230	<0.01
Entorhinal (mm^3^)	257	3803±650	422	3610±723	39	2919±705	<0.01
CMRgl	207	6.55±0.55	406	6.32±0.64	47	5.30±0.72	<0.01
SUVR	152	1.12±0.19	323	1.20±0.22	46	1.39±0.22	<0.01

NC, cognitively normal; MCI, mild cognition impairment; AD, Alzheimer's disease; CDR-SB, Clinical Dementia Rating sum of boxes; ADAS-cog, Alzheimer's disease Assessment Scale Cognition; MMSE, Mini-Mental State Exam; RAVLT, Rey Auditory Verbal Learning Test; FAQ, Functional Activities Questionnaire; CMRgl, Cerebral Metabolism Rate for glucose measured with fluorodeoxyglucose-positron emission tomography (FDG-PET). SUVR, florbetapir standard uptake value ratios on amyloid imaging.

* P values for continuous variables are from one-way analysis of variance (ANOVA). P values for categorical data are from chi square test.

Data are given as mean ± standard deviation unless otherwise indicated

### Impacts of *HMGCR* (rs3846662) on MRI structure

**Figure 1:**
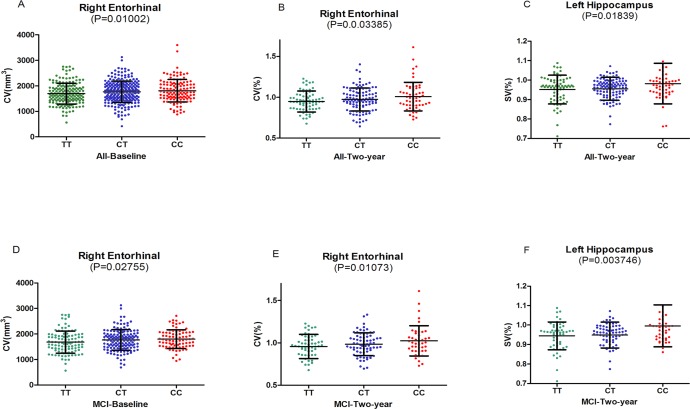
**A. The significant associations of *HMGCR* rs3846662 with baseline volume of right entorhinal in the hybrid population. B.** The significant associations of *HMGCR* rs3846662 with atrophy rate of right entorhinal in the hybrid population.**C.** The significant associations of *HMGCR* rs3846662 with atrophy rate of left hippocampus in the hybrid population. **D.** The significant associations of *HMGCR* rs3846662 with baseline volume of right entorhinal in the MCI group. MCI = mild cognition impairment. **E.** The significant associations of *HMGCR* rs3846662 with atrophy rate of right entorhinal in the MCI group. MCI = mild cognition impairment. **F.** The significant associations of *HMGCR* rs3846662 with atrophy rate of left hippocampus in the MCI group. MCI = mild cognition impairment.

Here we selected several cortical areas as the ROIs of the MRI measures analysis, including the volume of middle temporal gyrus, posterior cingulate, precuneus, parahippocampal gyrus, and hippocampus (Supplementary Table 1). In the cortical structure analysis, baseline volume of right entorhinal was identified to have strong associations with SNP rs3846662 in the hybrid population (the population included NC, MCI and AD patients) (*P* = 0.01002) (Figure 1A). Besides, patients with CC and CT genotypes had greater volume than those with TT genotypes in right entorhinal (CC: 1808±446.2 mm^3^, CT: 1764±408.2 mm^3^, TT: 1700±410.8 mm^3^, *P* = 0.01002) (Table 3) (Figure 1A). So it was detected that right entorhinal of C-allele carriers in the subjects showed significant prevention on the percentage of atrophy. Then we compared the volume between TT, CT, and CC groups in the three different clinical stages (NC, MCI, and AD). We observed that rs3846662 only prevent the atrophy of right entorhinal in the MCI group (*P* = 0.02755) (Figure 1D). In the subcortical structure analysis, we didn't find the baseline volume of any region have relationship with SNP rs3846662 in the hybrid population.

In the follow-up research of two years, we found that rs3846662 decrease the percentage of atrophy in the volume of right entorhinal (*P* = 0.03385) (Figure 1B). The C-allele in remarkably protected right entorhinal from shrinking at the two-year follow-up (CC: 0.99±0.1738, CT: 0.979±0.136, TT: 0.95±0.1323) (Table 3). Similarly, we can get the results in the MCI group (*P* = 0.01073) (Figure 1E). With regard to the subcortical structures, rs3846662 decreased the percentage of atrophy in the volume of left hippocampus in the hybrid population (*P* = 0.01839) (Figure 1C). We can get same results in both dominant and recessive pattern (Supplementary Table 2). The C-allele prevented left hippocampus atrophy (CC: 0.9828±0.1051, CT: 0.9564±0.06219, TT: 0.9515±0.07469, *P* = 0.01839) (Table 3). Furthermore, in the MCI cohort, the volume of left hippocampus was found to be related to rs3846662 (*P* = 0.003746) in the follow-up research of two years (Figure 1F). In addition, the C-allele prevented left hippocampus atrophy in MCI subjects. More importantly, *HMGCR* (rs3846662) was not found to be related to hippocampus or hippocampus substructure (CA1) (*P* > 0.5) (Supplementary Table 1), the most associated substructure with the AD specific amnestic syndrome in hippocampus [9].

### Impacts of *HMGCR* (rs3846662) on glucose metabolism

**Figure 2:**
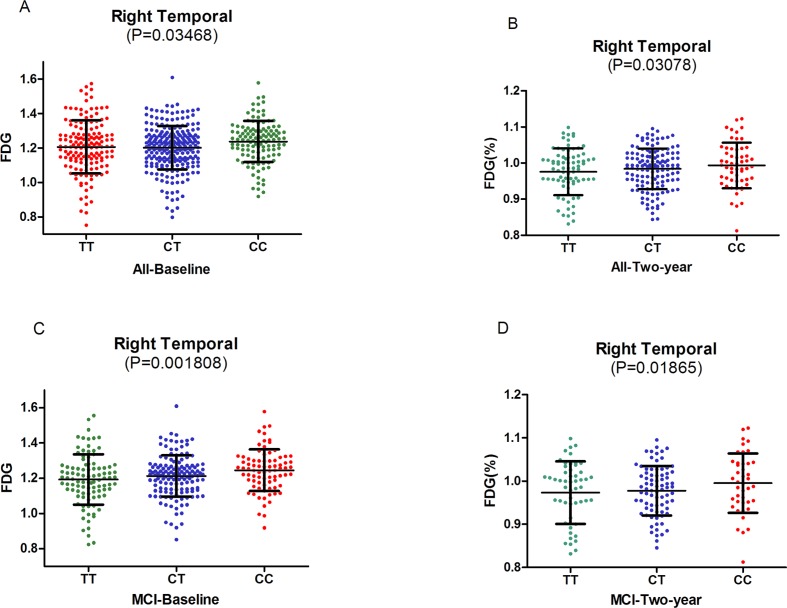
**A. The significant association of *HMGCR* rs3846662 with glucose metabolism of right temporal in the hybrid population. B.** The significant associations of *HMGCR* rs3846662 with glucose metabolism rate of right temporal in the hybrid population.**C.** The significant associations of *HMGCR* rs3846662 with glucose metabolism of right temporal in the MCI group. MCI = mild cognition impairment.**D.** The significant associations of *HMGCR* rs3846662 with glucose metabolism rate of right temporal in the MCI group. MCI = mild cognition impairment.

We test the relationships between *HMGCR* genotypes and the cerebral metabolism rate of glucose (CMRgl) on FDG-PET imaging (Supplementary Table 2). Right temporal was found to be related to rs3846662 at baseline in the hybrid population (*P* = 0.03468) (Figure 2A). In the analysis of the three groups, same result was found in MCI groups at baseline (*P* = 0.001808) (Figure 2C). The C-allele had significantly higher CMRgl in the right temporal (CC: 1.237±0.1166, CT: 1.212±0.1146, TT: 1.189±0.1413, *P* = 0.001808) (Table 2) (Figure 2C).

**Table 2:** The characteristics of included SNP

SNP	Position	Minor allele	MAF				Previous studied articles (PMID)
			ALL	AD	MCI	NC	
			0.462	0.49	0.47	0.445	18559695
Rs3846662	Intron variant	C	H-W (*p* value)				25023145 18802019
			ALL	AD	MCI	NC	20145341
			0.067	0.944	0.018	1.0	21867541

NC, normal control; MCI, mild cognition impairment; AD, Alzheimer's disease;

**Table 3:** Brain regions and regional changed PiB retention values on PET

	Regions		TT		CT		CC	*P*
**Baseline**	**ALL**	**N**	**Mean±SD**	**N**	**Mean±SD**	**N**	**Mean±SD**	
CV(mm3)	Right Entorhinal	163	1700±410.8	247	1764±408.2	126	1808±446.2	0.010
FDG	Right Temporal	172	1.20±0.1515	257	1.20±0.1232	136	1.23±0.1185	0.035
	MCI							
CV(mm3)	Right Entorhinal	104	1690±430.8	145	1762±389.2	83	1799±364	0.028
FDG	Right Temporal	111	1.189±0.1413	156	1.212±0.1146	92	1.237±0.1166	0.002
**Two years (%)**	**ALL**							
CV	Right Entorhinal	70	0.95±0.1323	113	0.979±0.136	55	0.99±0.1738	0.034
FDG	Right Temporal	94	0.972±0.06558	151	0.9845±0.05626	67	0.993±0.06115	0.031
SV	Left Hippocampus	70	0.9515±0.07469	113	0.9564±0.06219	55	0.9828±0.1051	0.018
	**MCI**							
CV	Right Entorhinal	52	0.9553±0.1455	72	0.9921±0.1282	44	1.016±0.182	0.011
FDG	Right Temporal	65	0.9731±0.07254	89	0.9791±0.05922	52	0.9965±0.06403	0.019
SV	Left Hippocampus	52	0.9473±0.07136	72	0.9503±0.07052	44	0.9962±0.1064	0.004

MCI, mild cognition impairment.

In the follow-up research of two years, rs3846662 may lower the drop rate of CMRgl in right temporal in the hybrid population (*P* = 0.03078) (Figure 2B). In the analysis of the three groups, we observed that rs3846662 may lower the drop rate of CMRgl in right temporal in MCI groups at the two-year follow-up (*P*= 0.01865) (Figure 2D). Besides, subjects carrying the C-allele had significantly higher CMRgl in the right temporal (CC: 0.9965±0.06403, CT: 0.9791±0.05922, TT: 0.9731±0.07254, *P* = 0.03078) (Table 3) (Figure 2D). However, we did not observe significant difference on other regional CMRgl in the other two groups (NC or AD).

### Impacts of *HMGCR* (rs3846662) on AV45-PET

In the analysis of AV45-PET, *HMGCR* (rs3846662) did not show an effect on florbetapir retention as well as the cortical SUVR. Likewise, we did not detect any significant associations on the changes over two years of these regions (Supplementary Table 3).

## DISCUSSION

Most brain cells generate cholesterol by two separate yet interrelated processes: synthesis by *HMGCR* and internalization of lipoproteins by means of the *APOE*/low density lipoprotein receptor cascade which is damaged in AD [4]. Cholesterol synthesis may be involved in AD neurodegeneration because of polymorphism in *HMGCR* was found to be related with AD [10]. Some variants of *HMGCR* have been identified as AD susceptibility loci, whereas little is known about the interaction between the *HMGCR* and risk of AD [2, 10]. Rs3846662 SNP in intron 13 of the *HMGCR* gene was found to significantly associate with sporadic AD [4]. This present study investigated the effects of *HMGCR* rs3846662 (TT, CT, and CC) on neuroimaging biomarkers in three clinical stages (NC, MCI, and AD).

It is recognized that regional brain atrophy could be an important underlying pathology in functional imaging studies of neurodegenerative diseases [11, 12]. A study has demonstrated that structural brain changes occur years before the clinical onset in AD and were localized to regions influenced by AD neuropathology [13]. Therefore, in our study we detected the influence of rs3846662 on different brain volumes. Our study showed that variants at rs3846662 would change the volume of right entorhinal in the two-year follow-up study and the C-allele remarkably prevented the atrophy of right entorhinal. Then the finding was confirmed by the stratified analyses of MCI group. In addition, rs3846662 had influence on the volume of left hippocampus in the two-year follow-up study and C-allele showed protective effect on the volume of left hippocampus. This was in accordance with previous MRI-based studies that hippocampal atrophy was a recognized biological marker of AD. The finding was confirmed by the stratified analyses of MCI group. But we did't found any relation in AD group due to the limited sample size. Apart from hippocampal, middle temporal has been implicated as a key brain region involved in the pathogenesis of AD [14, 15]. But we didn't confirm it in our study.

Cerebral glucose metabolic rate may be related with AD. We investigated the mechanism underlying the interaction between rs3846662 and AD in this study, and found that right temporal reveal higher CMRgl both at baseline and the two-year follow-up. In addition, in the stratified analyses of AD, MCI and NC group, we also discovered C-allele carriers increased CMRgl in right temporal in the follow-up study of two years.

Some previous studies have paid their attentions to the effects of cholesterol on Aβ metabolism. They have shown that brain cholesterol metabolism may play a certain role in AD development, with increased cellular cholesterol levels leading to high amyloid beta (Aβ) production [16-18]. Another study believe Aβ directly affects the activity of the HMGCR enzyme and the metabolism of cholesterol in neuronal cells, being γ-secretase dependent [8]. In our study, AV45-PET imaging was utilized to mark the presence and deposition of Aβ. Nevertheless, no significant effect was detected for rs3846662 SNP on florbetapir retention. In addition, in the stratified analyses of AD, MCI and NC group, we also didn't discover rs3846662 have connection with Aβ deposition. More evidence will be needed to illustrate the interactions between rs3846662 and Aβ deposition.

There are several potential limitations in our study. First, our results are obviously limited to the ADNI dataset and its small sample size. Second, a follow up of two years may be too short to detect the significant influence of *HMGCR* on the AD process, and further study need an increase of follow-up. Moreover, our results cannot represent other ethnicities, because our sample was restricted to Caucasians. Finally, unbalanced number of subgroups (AD, MCI and NC) also makes the conclusions of total cohort tends to the results arisen from MCI, which has the most case number. This might lead to untrue finding for the entire cohort. Hence, more studies are needed to perform to fill the gaps.

In general, it is possible that *HMGCR* (rs3846662) could be involved in the structural and functional modification of right entorhinal and left hippocampus throughout the AD physiopathological process. What's more, it influedced glucose metabolism of right temporal. *HMGCR* (rs3846662) plays an important role in AD-related neurodegenerative processes. Therefore, identification of specific factors that regulate *HMGCR* alternative splicing and elucidating the underlying mechanism may lead to a better understanding of its impact on regulating cellular cholesterol homeostasis and neurodegenerative processes. It is necessary to explore more clear understanding on the mechanisms in a larger sample, with longer follow-up and a wider range of people.

## MATERIALS AND METHODS

### Participants

Data used in this article were got from the ADNI database (www.loni.ucla.edu\ADNI). The ADNI is an ongoing, longitudinal, multicenter study aimed at developing clinical, genetic, serial magnetic resonance imaging (MRI), positron emission tomography (PET), and biochemical biomarkers to measure the progression of MCI and early AD [19]. ADNI is the result of efforts of many co-investigators in the National Institute on Aging, the National Institute of Biomedical Imaging and Bioengineering, the Food and Drug Administration, private pharmaceutical companies and nonprofit organizations and subjects have been recruited from over 50 sites across the United States and Canada [20]. The initial goal of ADNI was to recruit 800 subjects, but it has been followed by ADNI-GO and ADNI-2. To date, three protocols have recruited over 1500 adults, ages 55 to 90, to participate in the research, consisting of cognitively normal older subjects, people with early or late MCI, and people with early AD. For more information, see http://www.adni-info.org.

We enrolled participants according to criteria demonstrated in the ADNI study protocol (http://www.adni-info.org/scientists/adnistudyprocedures.aspx). The present analysis was restricted to participants whose genotype data of *HMGCR* single nucleotide polymorphisms (SNPs) were available. Furthermore, we selected only non-Hispanic (Caucasian) participants in order to avoid the population stratification effects which can lead to spurious findings. Finally, 812 individuals including 281 NC, 483 MCI (including 63 who converted to AD and 420 who did not) and 48 AD were included in our study. Baseline and longitudinal data including structural MRI and PET were collected as parts of this study. Furthermore, all participants underwent a series of baseline clinical tests including CDRSB, ADAS11, MMSE, ADAS13, RAVLT, FAQ.

### SNP selection and genotyping

We performed the quality control (QC) procedures using PLINK software, and the inclusion criteria were as follows: minimum call rates > 90%, minimum minor allele frequencies (MAF) > 0.01, Hardy-Weinberg equilibrium test *P* > 0.001. We only enrolled SNPs which have been studied, large case-control trials or other experimental studies. After quality control procedures, one tag SNP which captured the greatest amount of common variations in *HMGCR* (rs3846662) was remained for data analysis (Table 2).

### MRI structure

UCSF FreeSurfer datasets were used to conduct association test of *HMGCR* genotypes with brain structure and the cerebral image segmentation and analysis were performed with the FreeSurfer version 5.1 (http://surfer.nmr.mgh.harvard.edu/) based on the 2010 Desikan-Killany atlas [21]. This process mainly contained motion correction and averaging of multiple volumetric T1 weighted images (when more than one is available), removal of non-brain tissue using a hybrid watershed/surface deformation procedure, automated Talairach transformation, segmentation of the subcortical white matter and deep gray matter volumetric structures (including hippocampus, amygdala, caudate, putamen, ventricles) [22], intensity normalization, tessellation of the gray matter white matter boundary, automated topology correction, and surface deformation following intensity gradients to optimally place the gray/white borders at the location where the greatest shift in intensity defines the transition to the other tissue class. The technical details of these procedures are described in prior publications [23]. we selected regions have been reported to link to AD closely, such as hippocampus, parahippocampus, middle temporal, posterior cingulate, precuneus, and entorhinal cortex as our regions of interest (ROI) to analyze their associations with *HMGCR* genotypes [24-26].

### Glucose metabolism on imaging

FDG analysis data were from UC Berkeley and Lawrence Berkeley National Laboratory on the website (http://adni.loni.usc.edu/data-samples/access-data/) [27]. We extracted the mean counts about the five ROIs (left angular gyrus, right angular gyrus, bilateral posterior cingular, left inferior temporal gyrus, right inferior temporal gyrus) for each subject's FDG scans at baseline and 24-month. These images were spatially normalized in Statistical Parametric Mapping (SPM) to the MNI PET template. Then the intensity values were computed with SPM subroutines. Finally each ROIs mean were normalized by dividing it in pons/vermis reference region mean.

### AV45-PET

We obtained PET imaging data with amyloid tracer, florbetapir (AV-45), from UC Berkeley-AV45 analysis dataset on website (http://adni.loni.usc.edu/data-samples/access-data/). For AV45-PET, mean florbetapir uptake within 4 cortical regions (frontal, anterior/posterior cingulate, lateral parietal, and lateral temporal) was extracted. Cortical standardized uptake values ratios (SUVR) were calculated by averaging across the 4 cortical regions and dividing this average by whole cerebellum. Each mean florbetapir uptake of the 4 main regions and cortical SUVR were used for analysis.

### Statistical analysis

We used one-way analysis of variance (ANOVA) to test the differences in continuous variables. Categorical data were tested using chi-square test to analyze demographics and genotypic frequencies. In addition, ADNI samples were stratified into three groups (NC, MCI and AD) to detect the effects of *HMGCR* genetic variations on neuroimaging phenotypes in the three clinical stages respectively. Moreover, a multiple linear regression model which considered age, gender, education, and APOE ε4 status as covariates was used to estimate coefficients for testing possible correlation between various phenotypes and *HMGCR* genotypes. All statistical analyses were performed by R 3.12 and PLINK (http://pngu.mgh.harvard.edu/wpurcell/plink/).

## SUPPLEMENTARY MATERIAL TABLES



## References

[1] Jiang T, Yu JT, Tan L (2012). Novel disease-modifying therapies for Alzheimer's disease. Journal of Alzheimer's disease.

[2] Keller L, Murphy C, Wang HX, Fratiglioni L, Olin M, Gafvels M, Bjorkhem I, Graff C, Meaney S (2010). A functional polymorphism in the HMGCR promoter affects transcriptional activity but not the risk for Alzheimer disease in Swedish populations. Brain research.

[3] Medina MW, Gao F, Ruan W, Rotter JI, Krauss RM (2008). Alternative splicing of 3-hydroxy-3-methylglutaryl coenzyme A reductase is associated with plasma low-density lipoprotein cholesterol response to simvastatin. Circulation.

[4] Leduc V, De Beaumont L, Theroux L, Dea D, Aisen P, Petersen RC, Dufour R, Poirier J, Alzheimer's Disease Neuroimaging I (2015). HMGCR is a genetic modifier for risk, age of onset and MCI conversion to Alzheimer's disease in a three cohorts study. Molecular psychiatry.

[5] Hiura Y, Tabara Y, Kokubo Y, Okamura T, Goto Y, Nonogi H, Miki T, Tomoike H, Iwai N (2010). Association of the functional variant in the 3-hydroxy-3-methylglutaryl-coenzyme a reductase gene with low-density lipoprotein-cholesterol in Japanese. Circulation journal.

[6] Simmons CR, Zou F, Younkin SG, Estus S (2011). Evaluation of the global association between cholesterol-associated polymorphisms and Alzheimer's disease suggests a role for rs3846662 and HMGCR splicing in disease risk. Molecular neurodegeneration.

[7] Burkhardt R, Kenny EE, Lowe JK, Birkeland A, Josowitz R, Noel M, Salit J, Maller JB, Pe'er I, Daly MJ, Altshuler D, Stoffel M, Friedman JM, Breslow JL (2008). Common SNPs in HMGCR in micronesians and whites associated with LDL-cholesterol levels affect alternative splicing of exon13. Arteriosclerosis, thrombosis, and vascular biology.

[8] Licastro F, Porcellini E, Caruso C, Lio D, Corder EH (2007). Genetic risk profiles for Alzheimer's disease: integration of APOE genotype and variants that up-regulate inflammation. Neurobiology of aging.

[9] Sarazin M, Chauvire V, Gerardin E, Colliot O, Kinkingnehun S, de Souza LC, Hugonot-Diener L, Garnero L, Lehericy S, Chupin M, Dubois B (2010). The amnestic syndrome of hippocampal type in Alzheimer's disease: an MRI study. Journal of Alzheimer's disease.

[10] Recuero M, Vicente MC, Martinez-Garcia A, Ramos MC, Carmona-Saez P, Sastre I, Aldudo J, Vilella E, Frank A, Bullido MJ, Valdivieso F (2009). A free radical-generating system induces the cholesterol biosynthesis pathway: a role in Alzheimer's disease. Aging cell.

[11] Herrup K (2011). Commentary on “Recommendations from the National Institute on Aging-Alzheimer's Association workgroups on diagnostic guidelines for Alzheimer's disease. ” Addressing the challenge of Alzheimer's disease in the 21st century. Alzheimer's & dementia.

[12] He Y, Wang L, Zang Y, Tian L, Zhang X, Li K, Jiang T (2007). Regional coherence changes in the early stages of Alzheimer's disease: a combined structural and resting-state functional MRI study. NeuroImage.

[13] Tondelli M, Wilcock GK, Nichelli P, De Jager CA, Jenkinson M, Zamboni G (2012). Structural MRI changes detectable up to ten years before clinical Alzheimer's disease. Neurobiology of aging.

[14] Hayata TT, Bergo FP, Rezende TJ, Damasceno A, Damasceno BP, Cendes F, Stella F, Balthazar ML (2015). Cortical correlates of affective syndrome in dementia due to Alzheimer's disease. Arquivos de neuro-psiquiatria.

[15] Soldan A, Pettigrew C, Lu Y, Wang MC, Selnes O, Albert M, Brown T, Ratnanather JT, Younes L, Miller MI, Team BR (2015). Relationship of medial temporal lobe atrophy, APOE genotype, and cognitive reserve in preclinical Alzheimer's disease. Human brain mapping.

[16] Porcellini E, Calabrese E, Guerini F, Govoni M, Chiappelli M, Tumini E, Morgan K, Chappell S, Kalsheker N, Franceschi M, Licastro F (2007). The hydroxy-methyl-glutaryl CoA reductase promoter polymorphism is associated with Alzheimer's risk and cognitive deterioration. Neuroscience letters.

[17] Simons M, Keller P, De Strooper B, Beyreuther K, Dotti CG, Simons K (1998). Cholesterol depletion inhibits the generation of beta-amyloid in hippocampal neurons. Proceedings of the National Academy of Sciences of the United States of America.

[18] Rodriguez-Rodriguez E, Mateo I, Infante J, Llorca J, Garcia-Gorostiaga I, Vazquez-Higuera JL, Sanchez-Juan P, Berciano J, Combarros O (2009). Interaction between HMGCR and ABCA1 cholesterol-related genes modulates Alzheimer's disease risk. Brain research.

[19] Weiner MW, Veitch DP, Aisen PS, Beckett LA, Cairns NJ, Cedarbaum J, Green RC, Harvey D, Jack CR, Jagust W, Luthman J, Morris JC, Petersen RC, Saykin AJ, Shaw L, Shen L (2015). 2014 Update of the Alzheimer's Disease Neuroimaging Initiative: A review of papers published since its inception. Alzheimer's & dementia.

[20] Mueller SG, Weiner MW, Thal LJ, Petersen RC, Jack C, Jagust W, Trojanowski JQ, Toga AW, Beckett L (2005). The Alzheimer's disease neuroimaging initiative. Neuroimaging clinics of North America.

[21] Desikan RS, Segonne F, Fischl B, Quinn BT, Dickerson BC, Blacker D, Buckner RL, Dale AM, Maguire RP, Hyman BT, Albert MS, Killiany RJ (2006). An automated labeling system for subdividing the human cerebral cortex on MRI scans into gyral based regions of interest. NeuroImage.

[22] Fischl B, Salat DH, van der Kouwe AJ, Makris N, Segonne F, Quinn BT, Dale AM (2004). Sequence-independent segmentation of magnetic resonance images. NeuroImage.

[23] Jack CR, Bernstein MA, Fox NC, Thompson P, Alexander G, Harvey D, Borowski B, Britson PJ, J LW, Ward C, Dale AM, Felmlee JP, Gunter JL, Hill DL, Killiany R, Schuff N (2008). The Alzheimer's Disease Neuroimaging Initiative (ADNI): MRI methods. Journal of magnetic resonance imaging.

[24] Song Z, Insel PS, Buckley S, Yohannes S, Mezher A, Simonson A, Wilkins S, Tosun D, Mueller S, Kramer JH, Miller BL, Weiner MW (2015). Brain amyloid-beta burden is associated with disruption of intrinsic functional connectivity within the medial temporal lobe in cognitively normal elderly. The Journal of neuroscience.

[25] Killiany RJ, Hyman BT, Gomez-Isla T, Moss MB, Kikinis R, Jolesz F, Tanzi R, Jones K, Albert MS (2002). MRI measures of entorhinal cortex *vs* hippocampus in preclinical AD. Neurology.

[26] Teipel SJ, Pruessner JC, Faltraco F, Born C, Rocha-Unold M, Evans A, Moller HJ, Hampel H (2006). Comprehensive dissection of the medial temporal lobe in AD: measurement of hippocampus, amygdala, entorhinal, perirhinal and parahippocampal cortices using MRI. Journal of neurology.

[27] Landau SM, Harvey D, Madison CM, Reiman EM, Foster NL, Aisen PS, Petersen RC, Shaw LM, Trojanowski JQ, Jack CR, Weiner MW, Jagust WJ, Alzheimer's Disease Neuroimaging I (2010). Comparing predictors of conversion and decline in mild cognitive impairment. Neurology.

