# Supplementary material for: Effect of HMGCR genetic variation on neuroimaging biomarkers in healthy, mild cognitive impairment and Alzheimer's disease cohorts
Source: Oncotarget. 2016 Feb 29;7(12):13319–27. doi: 10.18632/oncotarget.7797 (PMC4924644; doi:10.18632/oncotarget.7797)
Supplement: Supplementary file 1 [file oncotarget-07-13319-s001.pdf]

Effect of HMGCR genetic variation on neuroimaging biomarkers in healthy, mild cognitive impairment and Alzheimer's disease cohorts

Supplementary Material

Supplementary Table 1: Association between *HMGCR* genetic variants and regional volume on MRI

SNP	Time	Regions	Minor allele	β	P	Test
rs3846662	baseline	left entorhinal	C	19	0.407	ADD
rs3846662	baseline	left middle temporal	C	45.62	0.525	ADD
rs3846662	baseline	left parahippocampal	C	12.39	0.547	ADD
rs3846662	baseline	left posterior cingulate	C	11.31	0.645	ADD
rs3846662	baseline	left precuneus	C	14.17	0.795	ADD
rs3846662	baseline	right entorhinal	C	59.73	0.010	ADD
rs3846662	baseline	right middle temporal	C	65.71	0.393	ADD
rs3846662	baseline	right parahippocampal	C	25.44	0.159	ADD
rs3846662	baseline	right posterior cingulate	C	9.072	0.698	ADD
rs3846662	baseline	right precuneus	C	68.36	0.226	ADD
rs3846662	2-year	left entorhinal	C	0.01154	0.389	ADD
rs3846662	2-year	left middle temporal	C	0.005014	0.319	ADD
rs3846662	2-year	left parahippocampl	C	0.005956	0.488	ADD
rs3846662	2-year	left posterior cingulate	C	0.003446	0.467	ADD
rs3846662	2-year	left precuneus	C	0.007553	0.179	ADD
rs3846662	2-year	right entorhinal	C	0.02781	0.034	ADD
rs3846662	2-year	right middle temporal	C	0.0001024	0.983	ADD
rs3846662	2-year	right parahippocampl	C	0.00926	0.201	ADD
rs3846662	2-year	right posterior cingulate	C	-0.0007125	0.902	ADD
rs3846662	2-year	right precuneus	C	0.004353	0.437	ADD
rs3846662	baseline	left amygdala	C	9.276	0.485	ADD
rs3846662	baseline	left hippocampus	C	-12.99	0.667	ADD
rs3846662	baseline	right amygdala	C	18.28	0.168	ADD
rs3846662	baseline	right hippocampus	C	17.74	0.555	ADD
rs3846662	2-year	left amygdala	C	0.00227	0.791	ADD
rs3846662	2-year	left hippocampus	C	0.01656	0.018	ADD
rs3846662	2-year	right amygdala	C	-0.004134	0.616	ADD
rs3846662	2-year	right hippocampus	C	0.006852	0.173	ADD
rs3846662	baseline	left CA1	C	-3.792	0.133	ADD
rs3846662	baseline	right CA1	C	-2.424	0.303	ADD
rs3846662	2-year	left CA1	C	0.007829	0.348	ADD
rs3846662	2-year	right CA1	C	0.00391	0.637	ADD
rs3846662	baseline	right entorhinal	C	87.37	0.017	DOM
rs3846662	2-year	right entorhinal	C	0.04427	0.033	DOM
rs3846662	2-year	left hippocampus	C	0.0177	0.022	DOM

rs3846662	baseline	right entorhinal	C	73.68	0.066	REC
rs3846662	2-year	right entorhinal	C	0.02961	0.186	REC
rs3846662	2-year	left hippocampus	C	0.02791	0.020	REC

NOTE: The significant P value is highlighted in red.

Supplementary Table 2: Association between *HMGCR* genetic variants and metabolism rate of glucose on FDG-PET imaging

SNP	Time	Regions	Minor allele	β	P	Test
rs3846662	baseline	left angular	C	0.01516	0.088	ADD
rs3846662	baseline	right angular	C	0.01782	0.034	ADD
rs3846662	baseline	bilateral posterior cingular	C	0.02088	0.028	ADD
rs3846662	baseline	left temporal	C	0.01653	0.053	ADD
rs3846662	baseline	right temporal	C	0.01527	0.035	ADD
rs3846662	2-year	left angular	C	0.003287	0.515	ADD
rs3846662	2-year	right angular	C	0.007442	0.141	ADD
rs3846662	2-year	bilateral posterior cingular	C	0.0036	0.419	ADD
rs3846662	2-year	left temporal	C	0.008502	0.091	ADD
rs3846662	2-year	right temporal	C	0.01033	0.031	ADD
rs3846662	baseline	right angular	C	0.02123	0.115	DOM
rs3846662	baseline	bilateral posterior cingular	C	0.02412	0.112	DOM
rs3846662	baseline	right temporal	C	0.01263	0.275	DOM
rs3846662	2-year	right temporal	C	0.01483	0.046	DOM
rs3846662	baseline	right angular	C	0.02808	0.053	REC
rs3846662	baseline	bilateral posterior cingular	C	0.03379	0.039	REC
rs3846662	baseline	right temporal	C	0.03052	0.014	REC
rs3846662	2-year	right temporal	C	0.01262	0.129	REC

NOTE: The significant P value is highlighted in red.

Supplementary Table 3: Association between *HMGCR* genetic variants and Aβ accumulation on AV-45 PET

SNPs	Time	Regions	Minor allele	β	P
rs3846662	baseline	cingulate	C	-0.02047	0.216
rs3846662	baseline	frontal	C	-0.02286	0.141
rs3846662	baseline	parietal	C	-0.01623	0.299
rs3846662	baseline	summarysuvr_wholecerebnorm	C	-0.01646	0.151
rs3846662	baseline	temporal	C	-0.02006	0.155
rs3846662	2-year	cingulate	C	-0.00207	0.755
rs3846662	2-year	frontal	C	-0.004118	0.527
rs3846662	2-year	parietal	C	-0.00222	0.726
rs3846662	2-year	summarysuvr_wholecerebnorm	C	-0.0023	0.595
rs3846662	2-year	temporal	C	-0.003028	0.613
